# SOAPTyping: an open-source and cross-platform tool for sequence-based typing for HLA class I and II alleles

**DOI:** 10.1186/s12859-020-03624-0

**Published:** 2020-07-08

**Authors:** Yong Zhang, Yongsheng Chen, Huixin Xu, Junbin Fang, Zijian Zhao, Weipeng Hu, Xiaoqin Yang, Jia Ye, Yun Cheng, Jiayin Wang, Weiqiang Sun, Jian Wang, Huanming Yang, Jing Yan, Lin Fang

**Affiliations:** 1 BGI-Shenzhen, Shenzhen, 518083 China; 2 Department of Biology, University of Copenhagen, Copenhagen, Denmark; 3 Geneplus-Beijing, Beijing, 102206 China; 4 BGI Genomics, Shenzhen, 518083 China; 5 China National GeneBank, BGI-Shenzhen, Shenzhen, 518120 China; 6 James D. Watson Institute of Genome Science, Hangzhou, 310008 China; 7 Zhejiang Hospital, No 12 Lingyin Road, Hangzhou, 310013 Xihu District China; 8 Department of Computer Science and Technology, Xi’an Jiaotong University, 28 West Xianning Road, Xi’an, 710048 Shaanxi China; 9 Shanghai Institute for Advanced Communication and Data Science, Shanghai Jiao Tong University, Shanghai, 200240 China

**Keywords:** HLA typing, Sequence-based typing, Sanger sequencing, Group specific sequencing primers

## Abstract

**Background:**

The human leukocyte antigen (HLA) gene family plays a key role in the immune response and thus is crucial in many biomedical and clinical settings. Utilizing Sanger sequencing, the golden standard technology for HLA typing enables accurate identification of HLA alleles in high-resolution. However, only the commercial software, such as uTYPE, SBT-Assign, and SBTEngine, and very few open-source tools could be applied to perform HLA typing based on Sanger sequencing.

**Results:**

We developed a user-friendly, cross-platform and open-source desktop application, known as SOAPTyping, for Sanger-based typing in HLA class I and II alleles. SOAPTyping can produce accurate results with a comprehensible protocol and featured functions. Moreover, SOAPTyping supports a more advanced group-specific sequencing primers (GSSP) module to solve the ambiguous typing results. We used SOAPTyping to analyze 36 samples with known HLA typing from the University of California Los Angeles (UCLA) International HLA DNA Exchange platform and 100 anonymous clinical samples, and the HLA typing results from SOAPTyping are identical to the golden results and 5.5 times faster than commercial software uTYPE, which shows the usability of SOAPTyping.

**Conclusions:**

We introduce the SOAPTyping as the first open-source and cross-platform HLA typing software with the capability of producing high-resolution HLA typing predictions from Sanger sequence data.

## Background

Human leukocyte antigens (HLA), encoded on 6p21.3, make up the human major histocompatibility complex (MHC) regions with high polymorphism and are featured in the immunity system [1]. Accurate HLA allele determination (‘HLA Typing’) is crucial in various biomedical and clinical processes, especially in the field of solid organ and bone marrow transplantation [2]. By January 2020, the database of the World Health Organization (WHO) Nomenclature Committee for Factors of the HLA System (IPD-IMGT/HLA Database) has collected 26,214 HLA alleles, including 19,031 HLA class I alleles containing HLA-A, −B, −C and -G genes, and 7183 HLA class II alleles covering HLA-DRB1, −DRB3, −DRB4, −DRB5, −DPA1, −DQA1, −DQB1 and -DPB1 genes [3, 4]. Among these alleles, HLA-A, −B, −C (class I), HLA-DRB1, −DQB1(class II) are relatively important and most commonly used for transplantation of hematopoietic. And Exons 2,3 for HLA class I genes, Exons 2 for HLA class II genes are designated as coding proteins involved in antigen presentation and are most commonly sequenced to determine high-resolution HLA types [4, 5].

Sequence-based typing (SBT), including Sanger sequence-based typing (SSBT) and next-generation sequence (NGS) typing, is widely used for high-resolution identification of HLA class I and II alleles [6]. Although NGS is advanced in sequencing throughput and cost and shows potential in rare HLA types discovery and higher resolution (up to four field allele resolution) HLA typing [7], the achievement of good allelic balance and homogenous coverage along all the target genes remains a major challenge [8, 9]. Moreover, erroneous and short reads produced by NGS also increase the complexity of bioinformatics algorithms in NGS-based HLA typing. A performance study of an NGS-based HLA typing method for clinical applications shows that the most frequent typing errors were caused by bioinformatics software [10]. To build capacity for NGS-based HLA typing method for clinical, elegant knowledge and skill in both laboratory technique and bioinformatics are highly required. On the other side, Sanger sequencing has its advantages in sequencing length and accuracy. SSBT has been widely used in the clinical laboratories since 1996 and still serves as the gold standard for HLA typing. Although, the heterozygous nature of SSBT method may give an ambiguous typing result for the combinations of many pairs of alleles [5, 11], a method called group-specific sequencing primers (GSSP) is adopted to enhance typing accuracy and can achieve a resolution of 99.9% of all SSBT ambiguities [11].

While SSBT is the golden standard technology for HLA typing for clinical use, there are no open-source tools currently available but only commercial and Windows-supported software, such as uTYPE (Life Technologies. Brown Deer, WI), SBT-Assign (Conexio, San Francisco, CA) and SBTEngine (GenDx, Utrecht, Netherlands), to perform sequence analysis and allele assignments for SSBT, and thus limits its application. Moreover, the escalating number of alleles significantly increased the percentage of ambiguous typing results and the numbers of possible allele pairs in each ambiguous typing [5]. As a result, the number of GSSPs had increased to around 300. A more intelligent function should be implemented to automatically and freely load all user-defined GSSPs and solve the ambiguous typing result, instead of dealing with the GSSPs one by one in uTYPE.

Hence, SOAPTyping was developed as a fast, accurate, and effective cross-platform software with a user-friendly interface for HLA class I and II typing using the SSBT method. Supported on Windows, Mac, and Linux, SOAPTyping also provides a neat and interactive user interface and generates a specialized report format. No proficient computer skills are required for users to effectively complete the analysis with a comprehensible protocol and produce accurate results. SOAPTyping also integrates a more intelligent GSSP prediction system to load all user-defined GSSPs in one operation. Moreover, SOAPTyping supports sample ID searching and can recover the analysis even when the program was shutdown. And theoretically, SOAPTyping can also be applied to other typing procedures if a proper reference sequence is provided. SOAPTyping is open source and freely available at https://github.com/BGI-flexlab/SOAPTyping. Users can also download the pre-compiled executables and databases for a different operating system from releases section on GitHub and run them directly.

### Implementation

#### Overview of SOAPTyping

SOAPTyping is a flexible and powerful application implemented in C++ with its user-friendly interface developed in the Qt framework, which is supported on Windows, Mac, and Linux. SOAPTyping is capable of analyzing loci located in HLA class I (A, B, C, and G) and II (DR-, DQ- and DP-) genes (Table 1). It mainly comprises of modules specialized for visualization, backend analysis, and database. The visualization module displays the samples, Sanger sequencing electropherograms, currently typing results, and interacts with the users to get the proper typing results by editing the wrong bases and solving ambiguous typing results. The backend analysis module performs base calling, alignment with the HLA database, and ambiguity solving with the GSSP method automatically after the proper actions at the visualization module. And the database module is used to store the HLA database, samples, and actions information that performed by the users. Together with the proposed best practices, users can easily and efficiently finish SSBT HLA typing in a short period.

**Table 1:** HLA molecules and the respective exon regions that can be analyzed by SOAPTyping

Genes	Exons
HLA-A	1,2,3,4,5,6
HLA-B	1,2,3,4,5
HLA-C	1,2,3,4,5,6,7
HLA-DRB1	1,2,3,4
HLA-DRB3,4,5	2,3
HLA-DQA1	1,2,3,4,
HLA-DQB1	1,2,3,4
HLA-DPB1	1,2,3,4
HLA-G	2,3,4
HLA-DPA1	1,2,3,4

#### Visualization

**Fig. 1:**
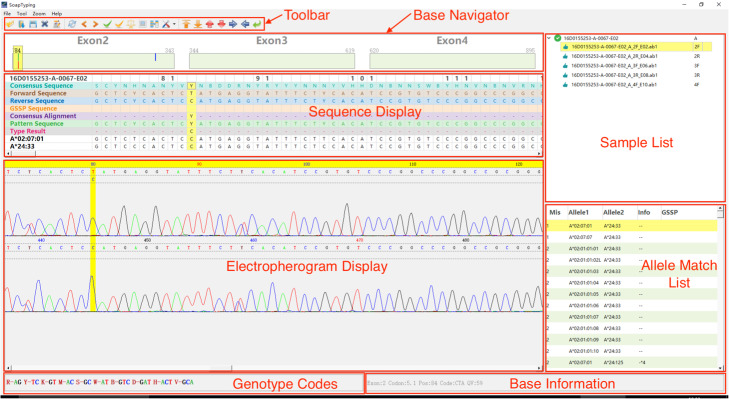
The main window of SOAPTyping. The panel of Sample List displays input files as a tree structure based on samples’ names and genes. The panel of Allele Match List displays possible typing results sorted by the number of mismatched sites. The panel of Base Navigator highlights mismatched positions so that users can skip to such positions quickly by clicking on the color bar. The panel of Sequence Display, from top to bottom, is comprised of server tracks including ‘Sample and Position’, ‘Consensus Sequence’, ‘Forward Sequence’, ‘Reverse Sequence’, ‘GSSP Sequence’, ‘Consensus Alignment’, ‘Pattern Sequence’, ‘Type Result’ and sequences of the allele pair. The panel of Electropherogram Display displays the electropherogram of the forward sequence, the reverse sequence, and the GSSP sequence so that users can edit bases in this area. The panel of Toolbar contains useful functions and information, such as importing and exporting reports

As shown in Fig. 1, the results are presented in the main window of SOAPTyping. The UI consists of panels of Toolbar, Base Navigator, Sequence Display, Sample List, Allele Match List, and Electropherogram Display. The functional descriptions of the interface are documented in the supplementary materials (Supplementary Section 1.1).

#### Backend analysis

The backend analysis module comprises three submodules, which are applied to perform base-calling from input electropherogram, HLA typing and GSSP module to deal with ambiguities. First, the base calling module is purposed to parse input electropherogram files to obtain base sequences. The HLA typing module aims to generate candidate allele pairs through aligning sequences to the consensus sequence of the IMGT/HLA database [6]. The GSSP sequences are leveraged to reduce ambiguities. Finally, all candidate allele pairs are collected and sorted according to the occurrences of mismatched sites.

##### Base calling submodule

Firstly, sequences derived from the input ABIF format [12] files are called homozygotes or heterozygotes. After the ABIF files are parallelly loaded to extract needed information, SOAPTyping obtains the details of base sequence, maximum signal position, quality values, and base signal values for each A/T/C/G base. To achieve the identification of heterozygotes and homozygotes, a peak range of each base is calculated using the following formulas. The *R*_*low*_ and *R*_*high*_ are the low and high range of the current base, *position*_*i*_ is the signal position of the current base peak, while *position*_*i* − 1_ and *position*_*i* + 1_ are the signal position of the previous and next base peak.

1 $$ {R}_{low}={position}_i-\frac{position_i-{position}_{i-1}}{2} $$

2 $$ {R}_{high}={position}_i+\frac{position_{i+1}-{position}_i}{2} $$

Secondly, SOAPTyping will search to find if there exists another peak within this range. If another peak exists with a signal value greater than 0.3 times the maximum signal within 4 units of distance, such a position will be determined as heterozygous genotypes. Homozygotes will be determined if only one peak exists within this range. The inferred genotypes are presented following the code standard of IUPAC-IUB.

##### HLA typing submodule

Being presented as lists of degenerate bases, sequences are aligned to the consensus sequences and alleles in the IMGT/HLA database to assign the eligible allele pairs using a modified semi-global alignment method. As the beginning or end of sequences may contain bases outside the exon regions, the semi-global alignment method does not penalize gaps at the beginning or end of the alignment. Another adjustment of our semi-global alignment method is that a comparison of one degenerated base will be considered between two independent alleles derived from that degenerated base, as shown in Formula 3. For example, comparisons between degenerated bases of A, R (AG), G, and reference A will end up with scores of 2, 1, and 0, respectively.

3 $$ \mathrm{Score}\left( seq{1}_i, seq{2}_j\right)=\left\{\begin{array}{c}2,\kern1.5em when\ 2\  allele s\ match\\ {}1,\kern0.5em when\ 1\  allele\ match\\ {}0,\kern7.25em mismatch\\ {}-1,\kern8.75em indel\end{array}\right. $$

Afterward, SOAPTyping will merge alignment results based on multiple input files. In the merging process, differences between forward and reverse sequences and those between sample sequences and IMGT/HLA types are stored in the dynamic database. Users can access the recorded differences at the Base Navigator of the main UI. Meanwhile, users can also edit mismatched bases at the pane of the Electropherogram Display Region, followed by SOAPTyping’s automated analysis repeatedly. Finally, SOAPTyping produces a standardized output with the nomenclature of HLA alleles [5].

##### GSSP submodule

GSSP is the widely accepted method to separately sequence one of the alleles, thus resolving the ambiguities. SOAPTyping supports not only the commercial GSSPs kits, such as SeCore™ Sequencing Kits (Invitrogen, Brown Deer, WI) but also the user-defined GSSP sequencing kits. First, these GSSPs should be imported to the database module, and SOAPTyping supports batch importing of all the GSSPs at a time, which is convenient for a large number of the GSSPs. Then, the GSSP sequences of each sample will be extracted, automatically identified, aligned to the HLA sequences, and used to handle the ambiguities. Users can combine the GSSP sequence results to manually filter the wrong HLA types and obtain the final type of the HLA alleles without ambiguity.

#### Database module

The databases in SOAPTyping are implemented using SQLite, which is a small, fast and reliable database engine. The database module mainly includes two kinds of databases, which are static and dynamic. Nucleotide sequence alignments as files of the IMGT/HLA database can be read by SOAPTyping directly, such files ending up being stored in the static database to serve as the reference of alignments. The GSSPs, only bounded to one of the two alleles present in the DNA sample, are also stored in the static database to support the determination of the final HLA typing. The involved database could be manually prepared for updates by following instructions in the supplementary materials (Supplementary Section 2.9). Meanwhile, there is also a dynamic database that stores intermediate data generated by the backend analysis module so that users could get back to states of former analysis even after they have shutdown SOAPTyping. The detailed designs of these database tables could be found in the Supplementary Section 1.2.

## Results

### Best practices / proposed workflow

**Fig. 2:**
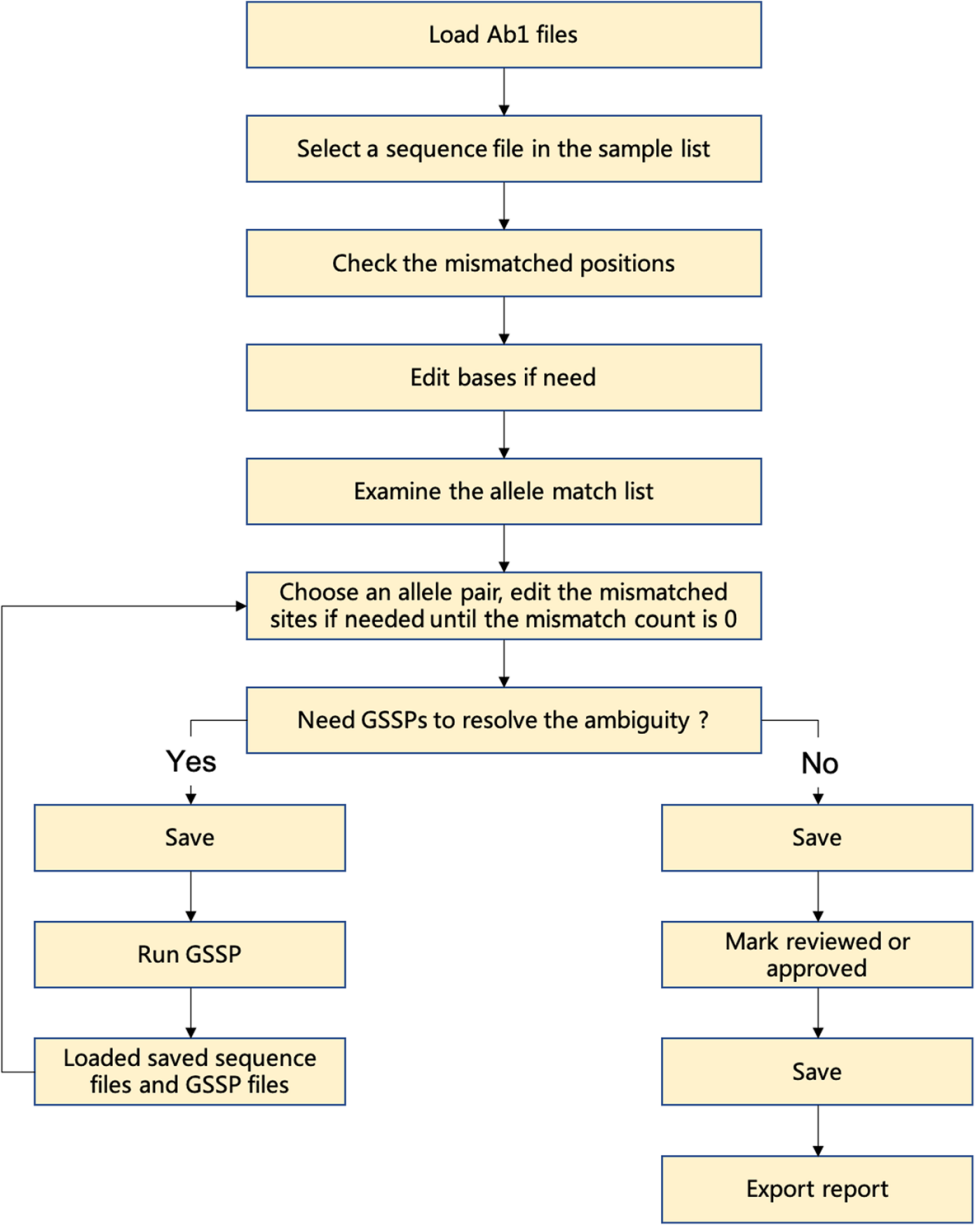
Best practices and proposed workflow for SOAPTyping

SOAPTyping works on chromatogram files with the format of ABIF, including .ab1 and .fsa files, which are generated from Sanger sequencing by ABI Genetic Analyzer Software (Applied Biosystems, Foster City, CA). Top candidate allele pair matches are presented in the Allele Match List. If necessary, users could manually review and edit marked positions that result from discrepant sites between forward and reverse sequences or mismatches with the consensus sequence(s) till completion of at least one trace with zero mismatches in the Allele Match List. If GSSP is needed to solve the ambiguities, the user can load GSSP sequences with pre-analyzed Exon sequences into SOAPTyping, solve the mismatches, and combine the results to get the unambiguous types. Best practices and proposed workflow are provided in Fig. 2 and Supplementary Section 2 to facilitate and guide the efficient use of SOAPTyping.

### Testing on UCLA samples and anonymous clinical samples

To verify the accuracy of SOAPTyping, our test data contains 36 samples initiated for external quality assessments with the University of California Los Angeles (UCLA) International HLA DNA Exchange (Los Angeles, CA, USA). Genomic DNAs with known HLA typing results were obtained from UCLA and amplified using locus-specific primers. The PCR products were directly sequenced in exons of HLA-A, −B, −C, −DRB1, and -DQB1 (Table S1) using a 3730XL DNA Analyzer (Applied Biosystems, Foster City, CA). The Sequencing reaction was performed using the BigDye® Terminator v3.1 Cycle Sequencing Ready Reaction Kit (Applied Biosystems). The sequence was analyzed with SOAPTyping and uTYPE, which are used in typing application in BGI, and the typing results were compared to the consensus-based on the high resolution provided by UCLA. The consistency of SOAPTyping in typing HLA alleles at two-field designations was verified to be accurate at the level of 100% (36/36) for HLA-A, 100% (36/36) for HLA-B, 100% (36/36) for HLA-C, and 100% (36/36) for HLA- DRB1, 100% (36/36) for HLA- DQB1. uTYPE also shows the same consistent results with SOAPTyping. The detailed results of 36 tested samples were shown in Table S8.

To further compare the performance of SOAPTyping and uTYPE in clinical, 100 anonymous clinical samples generated the same as the UCLA samples had been tested on a Thinkpad × 270 computer with Windows 10 system. The HLA typing results at two-field designations of SOAPTyping and uTYPE are identical at all sequenced genes (HLA-A, B, C, DRB1, DQB1). The detailed results of the 100 tested samples are list in Table S9. The analysis time had also been recorded in 10 samples/run (Table S10). The average analysis time of a sample is 8.43 s using SOAPTyping, while 46.38 s spent using uTYPE, which is about 5.5 times slower.

## Conclusions

Therefore, SOAPTyping is introduced in this article as the first open-source and cross-platform HLA typing software to our community with the capability of producing high-resolution HLA typing predictions from Sanger sequence data. Comparing to the commercial software, SOAPTyping is designed with a more advanced GSSP function to load a large number of GSPPs into the database at one time and automatically identify the GSSP sequences instead of the tedious manual operations. And with the design of the dynamic database, SOAPTyping can load massive samples into the workbench and can resume the analysis anytime after the SOAPTyping had been shutdown. As high-consistent HLA types with golden standard of UCLA samples are achieved and comparison with commercial software uTYPE shows SOAPTyping is 5.5 times faster with identical HLA typing results, we demonstrated that SOAPTyping could be efficiently and effectively applied to practical research and clinical use.

In future developments of the SOAPTyping, improvements of the efficiency of alignment algorithm for the candidate allele pairs are needed due to the challenges of upscaling of the HLA alleles in the IMGT/HLA database. Meanwhile, SOAPTyping can also be applied to support any kind of allele typing of Sanger sequencing data with fewer adjustments on the database and alignment algorithm according to the usage scenario.

### Availability and requirements

**Project name**: SOAPTyping.

**Project home page**: https://github.com/BGI-flexlab/SOAPTyping

**Operating system(s)**: Platform independent.

**Programming language**: C/C++, QT.

**Other requirements**: No.

**License**: GNU GPL.

**Any restrictions to use by non-academics**: No.

## Supplementary information

**Additional file 1. **SOAPTyping Supplement materials. **Table S1.** HLA molecules and the respective exon regions that can be analyzed by SOAPTyping. **Table S2.** Icons involved in the pane of Sample List. **Table S3.** Colors and their meanings in the pane of Base Navigator. **Table S4.** Detailed columns showed in the pane of Allele Match List. **Table S5.** Descriptions of each row in the pane of Sequence Display. **Table S6.** Descriptions of icons in the pane of Toolbar. **Table S7.** alleleTable. **Table S8.** gsspTable. **Table S9.** geneTable. **Table S10.** fileTable. **Table S11.** gsspFileTable. **Table S12.** sampleTable. **Figure S1.** The main window of SOAPTyping **Figure S2.** Best practices and proposed workflow for SOAPTyping **Figure S3.** Loading input file. **Figure S4.** An example exported report. **Figure S5.** Files required for database updates. **Figure S6.** The GSSP information window. **Figure S7.** Files required for GSSP database update. **Figure S8.** Allele alignment tool. **Table S8.** SOAPTyping results of 36 samples from UCLA International DNA Exchange. **Table S9.** The HLA typing results of 100 clinical samples. **Table S10.** The running time of 100 clinical samples.

## Data Availability

The UCLA HLA DNA samples can be obtained through application from website https://www.uclahealth.org/pathology/uic-hla-reference-programs. And the UCLA HLA datasets generated and analyzed during the current study are available in the CNSA (https://db.cngb.org/cnsa/) of CNGBdb with an accession code CNP0000512, ftp://ftp.cngb.org/pub/CNSA/CNP0000512. The 100 anonymous clinical samples and datasets are not publicly available.

## Abbreviations

HLA: Human leukocyte antigen
MHC: Major histocompatibility complex
PCR: Polymerase chain reaction
SBT: Sequence-Based Typing
SSBT: Sanger sequence-based typing
NGS: Next-generation sequence
GSSP: Group-specific sequencing primers
UCLA: University of California Los Angeles
