# Supplementary material for: SOAPTyping: an open-source and cross-platform tool for sequence-based typing for HLA class I and II alleles
Source: BMC Bioinformatics. 2020 Jul 8;21:295. doi: 10.1186/s12859-020-03624-0 (PMC7646500; doi:10.1186/s12859-020-03624-0)
Supplement: Supplementary file 1 — Additional file 1. SOAPTyping Supplement materials. Table S1. HLA molecules and the respective exon regions that can be analyzed by SOAPTyping. Table S2. Icons involved in the pane of Sample List. Table S3. Colors and their meanings in the pane of Base Navigator. Table S4. Detailed columns showed in the pane of Allele Match List. Table S5. Descriptions of each row in the pane of Sequence Display. Table S6. Descriptions of icons in the pane of Toolbar. Table S7. alleleTable. Table S8. gsspTable. Table S9. geneTable. Table S10. fileTable. Table S11. gsspFileTable. Table S12. sampleTable. Figure S1. The main window of SOAPTyping Figure S2. Best practices and proposed workflow for SOAPTyping Figure S3. Loading input file. Figure S4. An example exported report. Figure S5. Files required for database updates. Figure S6. The GSSP information window. Figure S7. Files required for GSSP database update. Figure S8. Allele alignment tool. Table S8. SOAPTyping results of 36 samples from UCLA International DNA Exchange. Table S9. The HLA typing results of 100 clinical samples. Table S10. The running time of 100 clinical samples. [file 12859_2020_3624_MOESM1_ESM.docx]

##

SOAPTyping Supplement materials

[1. Software architecture and function description 2](#_Toc41117298)

[1.1 Visualization module 2](#_Toc41117299)

[1.2 Database module 4](#_Toc41117300)

[2. Best practices / propositional workflow 6](#_Toc41117301)

[2.1 Load ABIF files 6](#_Toc41117302)

[2.2 Select sequence files in Sample List 7](#_Toc41117303)

[2.3 Check mismatched positions 7](#_Toc41117304)

[2.4 Examine the allele match list 7](#_Toc41117305)

[2.5 Save and import unfinished jobs 7](#_Toc41117306)

[2.6 GSSP prediction system 7](#_Toc41117307)

[2.7 Mark the sample analysis stat 8](#_Toc41117308)

[2.8 Export reports 8](#_Toc41117309)

[2.9 Database update 8](#_Toc41117310)

[2.10 Utilities 10](#_Toc41117311)

[3. Verification using test data 10](#_Toc41117312)

[3.1 Test data 10](#_Toc41117313)

[3.2 Results 11](#_Toc41117314)

## Software architecture and function description

SOAPTyping is capable of analyzing loci located in HLA class I (A, B, C, and G) and II (DRB1, DRB3, DRB4, DRB5, DPA1, DQA1, DQB1, and DPB1) genes (Table S1). SOAPTyping includes three modules as illustrated in Figure S1, which are modules for (i) visualization, (ii) backend analysis, and (iii) database. These modules are described in further detail in the following part.

Table S1. HLA molecules and the respective exon regions that can be analyzed by SOAPTyping

| **Genes** | **Exons** | **Exons in the test data** |
| --- | --- | --- |
| HLA-A | 1,2,3,4,5,6 | 2,3,4 |
| HLA-B | 1,2,3,4,5 | 2,3,4 |
| HLA-C | 1,2,3,4,5,6,7 | 2,3,4 |
| HLA-DRB1 | 1,2,3,4 | 2,3 |
| HLA-DRB3,4,5 | 2,3 | NA |
| HLA-DQA1 | 1,2,3,4, | NA |
| HLA-DQB1 | 1,2,3,4 | 2,3 |
| HLA-DPB1 | 1,2,3,4 | NA |
| HLA-G | 2,3,4 | NA |
| HLA-DPA1 | 1,2,3,4 | NA |

### 1.1 Visualization module

The visualization module is designed to create an interactive interface, including multiple panes of SOAPTyping’s main window. The main window consists of panes of Sample List, Base Navigator, Allele Match List, Sequence Display Region, Electropherogram Display Region, and Toolbar (Figure S1).


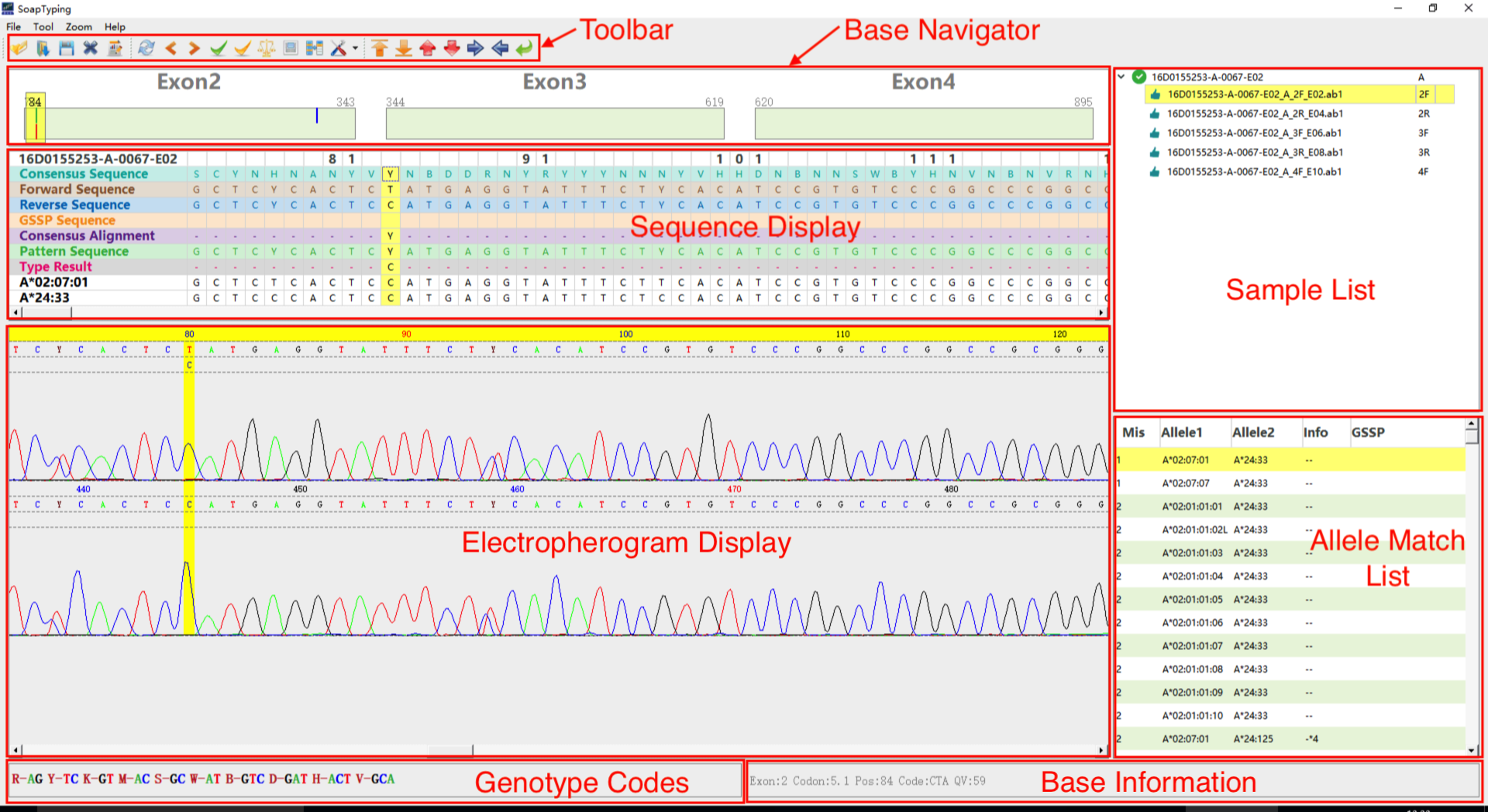


Figure S1. The main window of SOAPTyping.

- - 1. The pane of Sample List organizes the input files into a tree structure based on the sample name, in which nomenclature is given in the following Section 2.1. Involved icons and their detailed explanations are shown in Table S2.

Table S2. Icons involved in the pane of Sample List

| Icons | Descriptions |
| --- | --- |
| 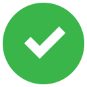 | No mismatches exist between pattern sequence and the allele pair. The allele pair is a common type. |
| 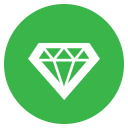 | No mismatches exist between pattern sequence and the allele pair. The allele pair is a rare type. |
| 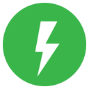 | No mismatches exist between pattern sequence and the allele pair. The quality of the sequence file is poor. |
| 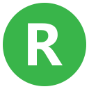 | No mismatches exist between pattern sequence and the allele pair. The sequence file is marked as reviewed. |
| 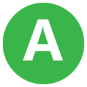 | No mismatches exist between pattern sequence and the allele pair. The sequence file is marked as approved. |
| 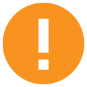 | Mismatches exist between pattern sequence and the allele pair. |
| 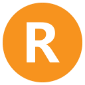 | Mismatches exist between pattern sequence and the allele pair. The sequence file is marked as reviewed. |
| 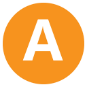 | Mismatches exist between pattern sequence and the allele pair. The sequence file is marked as approved. |
| 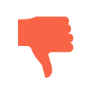 | The quality of the sequence file is poor. |
| 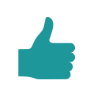 | The quality of the sequence file is satisfactory. |
| 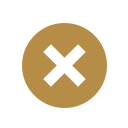 | The sequence file cannot be analyzed. |
| 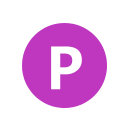 | The sequence file is marked as pending. |

- - 1. The pane of Base Navigator highlights mismatched positions so that users can skip to such positions quickly by clicking on the color bar, in which detailed descriptions are presented in Table S3. The pane of Base Navigator could be divided into two parts. The upper area displays the difference between the forward and backward sequence as well as the discrepancies between the pattern and consensus sequence. In the meantime, it also tracks the edited or filtered position. The lower area shows mismatches between the pattern sequence and its best-matched allele, which are only displayed when a specific allele pair is chosen.

Table S3. Colors and their meanings in the pane of Base Navigator

| Color | Descriptions |
| --- | --- |
| Blue | The forward and backward sequences are compatible but different. |
| Red | The pattern sequence is not compatible with the consensus sequence and there exist mismatches between pattern sequence and the chosen allele pair. |
| Green | The forward and backward sequences are not compatible. |
| Black | The base has been edited. |

- - 1. The pane of Allele Match List displays possible typing results sorted following the order of the number of mismatched sites. Detailed explanations of designed columns are listed in Table S4.

Table S4. Detailed columns showed in the pane of Allele Match List

| Columns | Descriptions |
| --- | --- |
| Mis | The number of mismatches between the allele pair and the pattern sequence. Note that ‘*’ indicates indel(s) exist(s) among at least one of the typed alleles. Such candidate allele pairs could be removed by a double-click. |
| Allele1 | The first candidate allele |
| Allele2 | The second candidate allele |
| Info | Information about allele pairs. A single “r” shows one of the candidate alleles is a rare type and thus double “r” means both candidate alleles are rare types. Symbol of “-” indicates no missing exons used to analyze in candidate alleles. Symbol of “*” followed with a number indicates a specific exon region is missing in the candidate allele. |
| GSSP | “Yes” means GSSP information is available |

- - 1. The pane of Sequence Display (Table S5), from top to bottom, is comprised of several tracks including ‘Sample and Position’, ‘Consensus Sequence’, ‘Forward Sequence’, ‘Reverse Sequence’, ‘GSSP Sequence’, ‘Consensus Alignment’, ‘Pattern Sequence’, ‘Type Result’ and sequences of the allele pair.

Table S5. Descriptions of each row in the pane of Sequence Display

| Rows | Descriptions |
| --- | --- |
| 1 | Sample name listed at the beginnings, followed by the position information |
| 2 | Consensus Sequence showing the consensus sequence of the locus being analyzed. |
| 3 | Forward sequences that are parsed from the input electropherogram files. |
| 4 | Reverse sequences that are parsed from the input electropherogram files. |
| 5 | GSSP sequences that are parsed from the input electropherogram files. |
| 6 | Consensus Alignment showing alignment results of the pattern sequence and the consensus sequence. |
| 7 | Pattern Sequence showing the combination of the forward and reverse sequence. For example, if a site in the forward sequence is ‘A’, the corresponding site in the reverse sequence is ‘R’, which means this site is a heterozygous site of ‘A’ and ‘G’, in which case the combination of this site is ‘R’. |
| 8 | Type results showing different alleles compared to the chosen allele pair in the pane of Allele Match List. |
| 9 | The sequence of the first allele of the chosen candidate allele pair in the pane of Allele Match List |
| 10 | The sequence of the second allele of the chosen candidate allele pair in the pane of Allele Match List |

- - 1. The pane of the Electropherogram Display Region (Figure S2) displays the electropherogram of the forward sequence, the reverse sequence, and the GSSP sequence, so that users can edit bases in this region.
    2. The pane of Toolbar integrates some useful functions and information. The detailed function of each icon is listed in Table S6. Corresponding degenerate bases, as well as signal information of the chosen base, are shown at the bottom of the UI.

Table S6. Descriptions of icons in the pane of Toolbar

| Icons | Descriptions |  |
| --- | --- | --- |
| **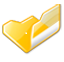** | Open new files |  |
| **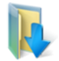** | Load saved files |  |
| **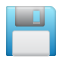** | Save |  |
| **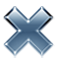** | Delete file in the pane of Sample List |  |
| **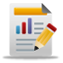** | Export the final report |  |
| **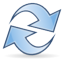** | Reset the file as initial status |  |
| **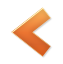** | Jump to the previous mismatched locus |  |
| **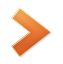** | Jump to the next mismatched locus |  |
| **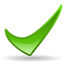** | Set all files status as “Approved” |  |
| **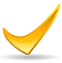** | Set all files status as “Reviewed” |  |
| **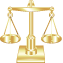** | Allele pair alignments |  |
| **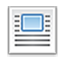** | Multiple alleles alignment |  |
| **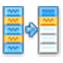** | Update databases |  |
| **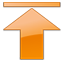** | Y range zoom of the electropherogram (+) | |
| **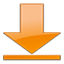** | Y range zoom of the electropherogram (-) | |
| **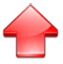** | Heighten the electropherogram | |
| **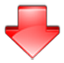** | Shorten the electropherogram | |
| **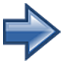** | Widen the electropherogram | |
| **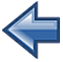** | Narrow the electropherogram | |
| **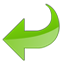** | Reset the electropherogram | |

### Database module

The database module mainly includes two kinds of databases, which are static and dynamic. The static database contains information that is not changed during runtime of SOAPTyping, which is composed of tables designed to store details related to HLA loci, alleles, exons, genes, and GSSPs. The table of loci stores consensus sequence and available exons that can be analyzed for each locus. The table of alleles stores HLA-I and HLA-II allele sequences and particular details of each allele, including rare variants and indels. The table of GSSP information table stores the GSSPs made for wanted exon regions, inserted sites, and inserted bases. These tables could be manually updated (see below at Section 2.9 for database updates). The dynamic database also applies different tables to store intermediate data generated during runtime, including tables of files, GSSP, and samples. The dynamic tables of files and GSSPs store information that are required to construct a sequence electropherogram. The table of sample mainly stores sequences parsed from input files and typing results of each sample.

***1.2.1 Static database***

Table S7. alleleTable

| **alleleName** | Store the allele name (eg: A*01:01:01:01) |
| --- | --- |
| **alleleSequence** | Store the allele sequence |
| **geneName** | Store the gene name |
| **isRare** | Store the result of rare type (0: normal 1:rare) |
| **isIndel** | Store the result of indel (0:normal 1:indel) |
| **indelPosition** | Store the indel position |
| **indelInfo** | Store the information of indel(eg: Delete 'N') |

Table S8. gsspTable

| **gsspKey** | Store gssp key which consists of gsspName, _,geneName |
| --- | --- |
| **gsspName** | Store gssp name |
| **geneName** | Store gene name |
| **exonIndex** | Store exon index |
| **rOrF** | Store the direction |
| **position** | Store the gssp position |
| **base** | Store the base of gssp position |

Table S9. geneTable

| **geneName** | Store gene name |
| --- | --- |
| **geneSequence** | Store gene sequence |
| **exonCount** | Store the exon count |
| **exonPositionIndex** | Store the exon pos array(eg: 0:73:343:619:895:1012:1045:1093:1098) |
| **geneClasses** | Store the gene class(eg: 0:192:194:224:226:240:242) |
| **availableExon** | Store the exon regions (eg: 123456) |

***1.2.2 Dynamic database***

Table S10. fileTable

| **fileName** | Store the name about the input ab1 file |
| --- | --- |
| **sampleName** | Store the sample name about the input ab1 file |
| **geneName** | Store the gene name about the input ab1 file |
| **exonIndex** | Store the exon index about the input ab1 file |
| **usefulSequence** | Store the alignment sequence about the input ab1 file |
| **baseSequence** | Store the original sequence about the input ab1 file |
| **basePosition** | Store the original position about the input ab1 file |
| **baseQuality** | Store the original quality about the input ab1 file |
| **baseASignal** | Store the original A signal about the input ab1 file |
| **baseTSignal** | Store the original T signal about the input ab1 file |
| **baseGSignal** | Store the original G signal about the input ab1 file |
| **baseCSignal** | Store the original C signal about the input ab1 file |

Table S11. gsspFileTable

| **fileName** | Store the name about the input gssp file |
| --- | --- |
| **sampleName** | Store the sample name about the input gssp file |
| **geneName** | Store the gene name about the input gssp file |
| **exonIndex** | Store the exon index about the input gssp file |
| **usefulSequence** | Store the alignment sequence about the input gssp file |
| **baseSequence** | Store the original sequence about the input gssp file |
| **basePostion** | Store the original position about the input gssp file |
| **baseQuality** | Store the original quality about the input gssp file |
| **baseASignal** | Store the original A signal about the input gssp file |
| **baseTSignal** | Store the original T signal about the input gssp file |
| **baseGSignal** | Store the original G signal about the input gssp file |
| **baseCSignal** | Store the original C signal about the input gssp file |

Table S12. sampleTable

| **sampleName** | Store the name about sample |
| --- | --- |
| **geneName** | Store the gene name about sample |
| **consensusSequence** | Store the consensus sequence which form genetable |
| **forwardSequence** | Store the forward sequence which form filetable |
| **reverseSequence** | Store the reverse sequence which form filetable |
| **patternSequence** | Store the pattern sequence which merge forward sequence and reverse sequence |

## Best practices / propositional workflow


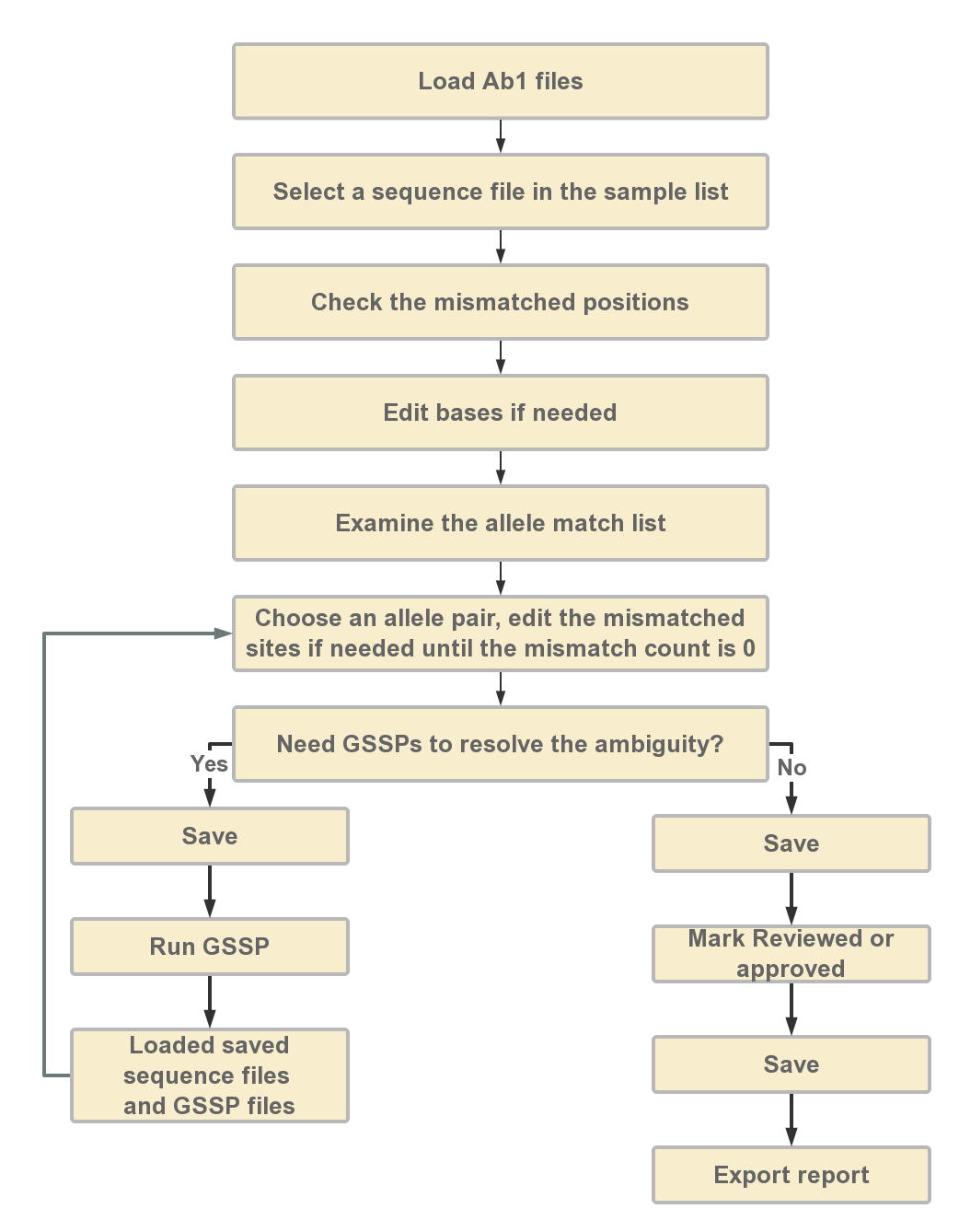
The best practices and propositional workflow are demonstrated in Figure S2. The details of each step are described below.

Figure S2. Best practices and proposed workflow for SOAPTyping.

### 2.1 Load ABIF files

Users can click the “open new file” icon at the toolbar to load the input files. During the loading process, information on imported files is shown in the ‘Open File’ window (Figure S3). To utilize the automatic extraction function, the file name should formatted as: [sample name]_[gene]_[exon region and strand direction]_[sequencer ID] (e.g., RB16002353_A_2R_A02 for sample name: RB16002353, gene: A, exon: 2, strand direction: Reverse, sequencer id: A02). For GSSP file, the file name should formatted as: [sample name]_[gene]_[GSSP name]_[sequencer ID]. If ambiguous nominations of input files cause improper information extraction, users can modify the names of input files by choosing correct information from the corresponding drop-down list. Users can also delete the files by choosing the file in front of file details and click the “Delete Chosen” button. After reassuring files information, users can perform the analysis by click the “Analysis” button.


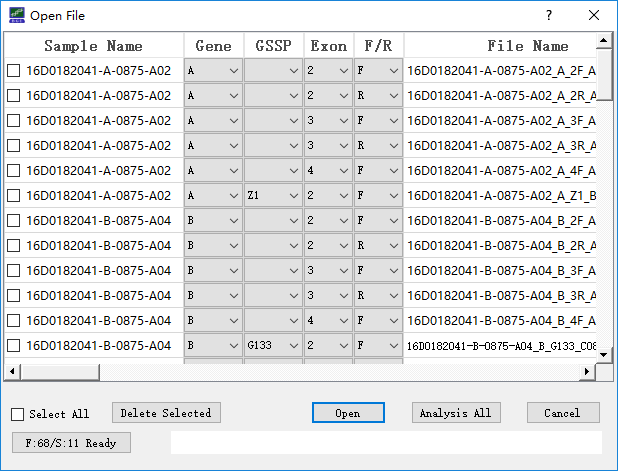


Figure S3. Loading input file.

### Select sequence files in Sample List

Afterward, imported sequence files are integrated based on sample name and gene, and displayed in the pane of Sample List. If a sample is selected, sequence information, electropherogram and candidate allele pairs of this sample will be displayed in the corresponding region of the main window (Figure S2).

### 2.3 Check mismatched positions

Two kinds of mismatch, compatible and non-compatible mismatches, are considered in SOAPTyping. For example, ‘R’ (heterozygotes of ‘A’ and ‘G’) and ‘A’ are compatible mismatches and ‘A’ and ‘G’ are non-compatible mismatch. Users need first to disambiguate the non-compatible sites in the forward and reverse sequence and then disambiguate the non-compatible sites in pattern sequence and consensus sequence. These mismatched sites are pointed out in the upper area of the pane of Base Navigator. Users can skip to those positions by clicking on the indication bar and edit bases if needed after examining the electropherogram. To edit a base, click on the site on the electropherogram and input the correct base. Once a base is edited, SOAPTyping will reanalyze this sample and update the allele match list and skip to the next mismatched position automatically. If users would like to perform analysis after all mismatched bases are edited, choose ‘Edit Multi’ from the ‘Zoom’ list.

### 2.4 Examine the allele match list

By selecting a suitable allele pair in the pane of the Allele Match List, the corresponding sequences of this allele pair will be shown in the pane of the Sequence Display Region. Mismatched sites between pattern the sequence and allele pair sequences are highlighted in red in ‘Type Result’ row and shown in the lower area in the pane of Base Navigator. Usually, there are one or serval allele pairs matched to the pattern sequence without any mismatches. But ~0.5% samples may not have a perfect match between the pattern sequence and allele pair sequences in the HLA databases due to the new types or other reasons. Users should make sure the combination of allele pair sequences is perfectly matched to pattern sequence before generating results.

### 2.5 Save and import unfinished jobs

SOAPTyping offers two solutions to save the results. One solution is to save the results of an individual sample by a right-click on this sample in the pane of the Sample List and then choosing options from “Quick Save”, “Quick Save and Clear” or “Quick Save by Date”. Another solution is clicking on the “Save” button in the toolbar to save the analysis outcome of multiple samples. During the saving process, a “Result” directory will be created in the working directory for the first time. Under the “Result” directory, each sample is assigned a subdirectory to save the original sequence files and intermediate data that are produced during analysis. For subsequent analysis, users can import saved files by clicking on the “Load” button (Figure S3). SOAPTyping will then search all analysis files of the day and import them, in which the search scope also can be determined by date and names of ABIF files.

### 2.6 GSSP prediction system

GSSP prediction system is used to resolve ambiguity, in which ambiguity means there are multiple candidate allele pairs that have no mismatches. To use a new GSSP, users need to add it to the database (see section 2.9 for database updates), otherwise, SOAPTyping cannot recognize it. Suitable GSSPs are found in SOAPTyping if an allele pair is marked as ‘Yes’ in the ‘GSSP’ column in the pane of the Allele Match List. To check GSSP information, users could right-click on the allele pair and choose ‘Show GSSP Z Code’. After resequencing to obtain ABIF files of GSSPs, users could import corresponding sequence files applied to resolve the ambiguity. SOAPTyping will search the candidate allele pairs for each GSSP file and users need to choose a suitable allele pair and edit bases if mismatched sites exist. Finally, users could click on ‘Combined’ under the sample will show the ultimate HLA typing result after all GSSP files have been examined.

### 2.7 Mark the sample analysis stat

Once sequence files of a sample are imported, SOAPTyping will automatically mark the status of the samples based on the sequencing quality of input files and match conditions of pattern sequence and candidate allele pairs. An icon indicating current status is shown before the sample name in the pane of the Sample List. Users can also mark the sample as ‘Pending’, ‘Reviewed’, and ‘Approved’ under accordance with the analysis progress. When a defective sample comes up or analysis is not complete, it may be suitable to mark the sample as ‘Pending’ for subsequent analysis. If the typing results are reviewed, such a sample can be marked as ‘Reviewed’. If typing results are affirmed, such a sample can be labeled as ‘Approved’. The status also can be canceled by choosing ‘Unlock’. All samples can be marked as ‘Reviewed’ or ‘Approved’ simultaneously by clicking on the corresponding button in the toolbar while marking ‘Pending’ is only allowed for a single sample.

### 2.8 Export reports

To export reports, users could click on the corresponding button in the pane of Toolbar and the window of the ‘Produce report’ will appear. Users can name reports and the output directory. When ‘Ignore Indel’ is selected, alleles that contain indels would be excluded. The ‘Allele Count’ option is to control numbers of the output alleles, otherwise, all alleles without mismatches will be exported by default. A report example is demonstrated in Figure S4.


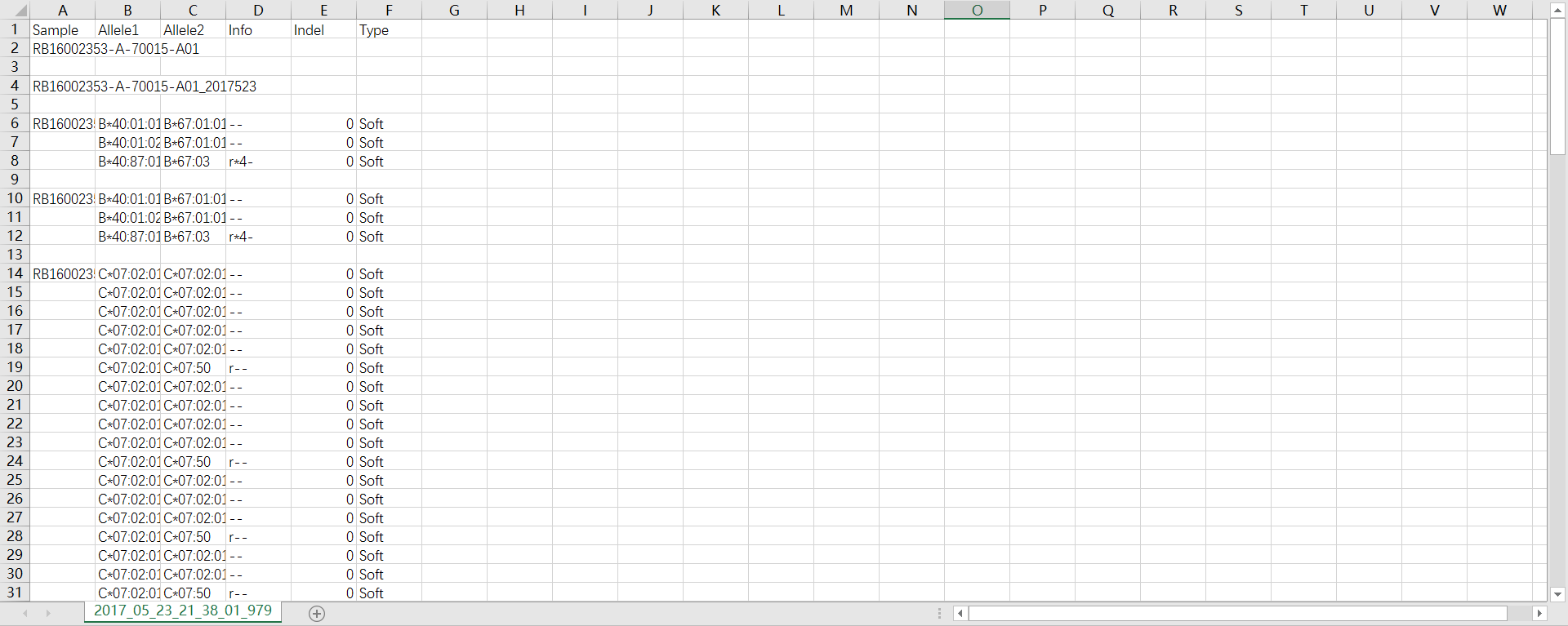


Figure S4. An example exported report. In the ‘Type’ column, ‘Soft’ indicates no GSSP is used and ‘Filter’ means the opposite.

### 2.9 Database update

SOAPTyping offers database updates function to cater to the situation of frequent updates of HLA alleles. Before updating the database, users could download the latest release of compressed alignments file (‘zip’ format, e.g., Alignments_Rel_3260.zip) of the IMGT/HLA database via its FTP directory (ftp://ftp.ebi.ac.uk/pub/databases/ipd/imgt/hla/). After decompressing the downloaded zip files, users can get an ‘alignments’ folder which contains genomics sequence alignments, nucleotide sequence alignments, and protein sequence alignments files for each locus. Then users can select the ‘alignments’ folder using the ‘Browser’ button on the right of the ‘All Nuc File:’ in the database update window (Figure S5).


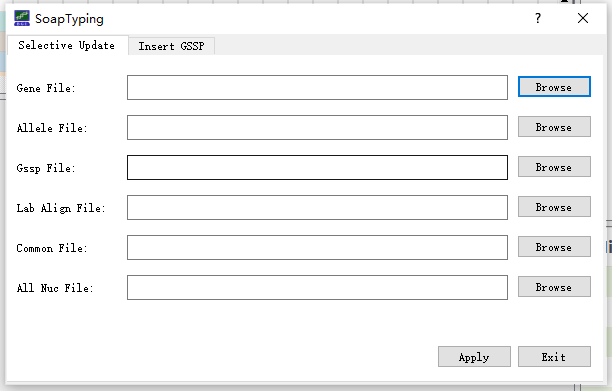


Figure S5. Files required for database updates

Additionally, if users want to update the GSSP information, the text file contains GSSP information should be prepared manually, in which examples are illustrated in Figure S6. Another way to update GSSPs is to fill the GSSP information in the corresponding fields in the window of ‘Insert GSSP’ (Figure S7).


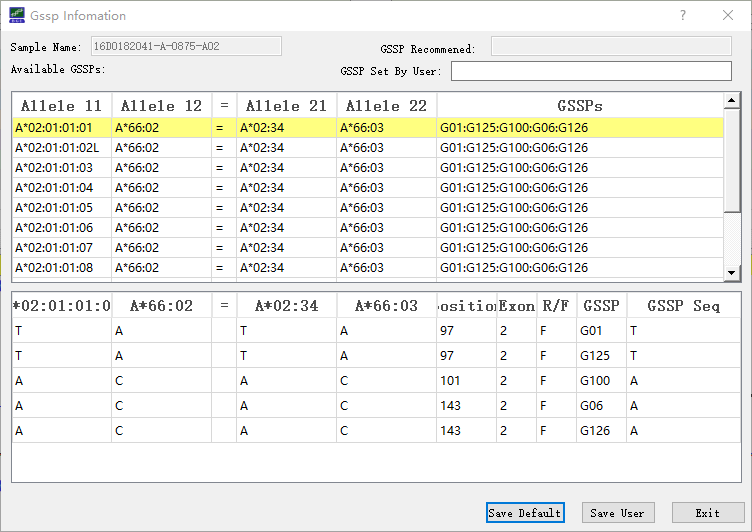


Figure S6. The GSSP column shows the GSSPs that can discern the ambiguity and the detailed information is shown below.


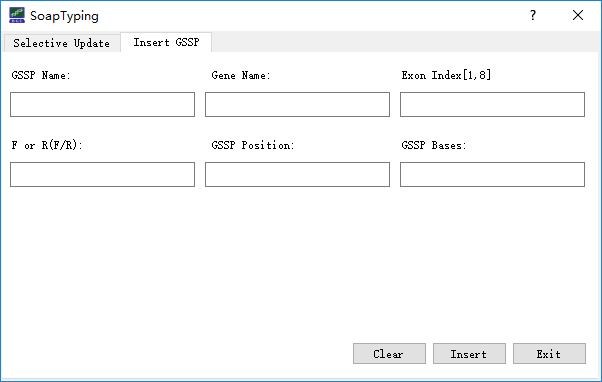


Figure S7. Files required for GSSP database update

### 2.10 Utilities

SOAPTyping supplies a set of utilities to assist HLA typing, which are listed as follows. (1) Setting analysis threads. Click on the ‘Setting’ button (Figure S1) and choose ‘Set Thread’ to set a suitable number of threads to accelerate analysis. (2) Customizing the exon region for analysis. Click on the ‘Setting’ button and choose ‘Set Exon Trim’ to exclude the bases of both ends of the exon, in which excluded parts are not considered in the analysis. (3) Alleles alignment (Figure S8a). Click on the ‘Allele Alignment’ button to invoke the alignment tool and choose a locus from the ‘Genes’ drop-down list. All available alleles of this locus will be listed in the panel below (Figure S8b). The alignment result will be displayed after the alleles of interest are chosen.


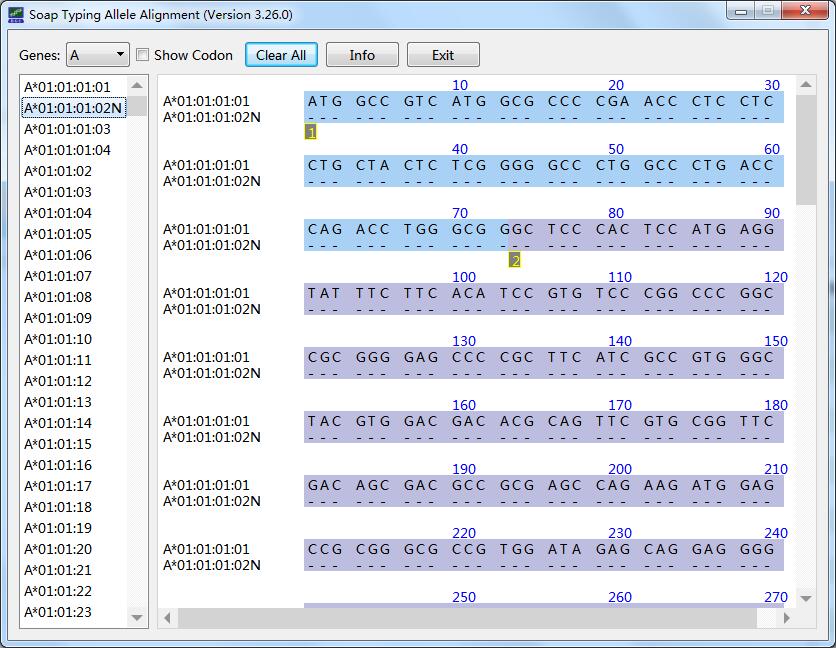

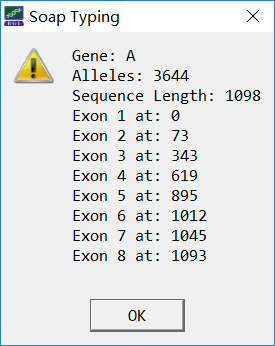


1. b)

Figure S8. Allele alignment tool. a) Alleles of a locus will be aligned to a standard sequence. ‘-’ means a base is correctly matched, otherwise, mismatched bases are shown. ‘*’ indicates a base is missing. b) Clicking ‘Info’ will display the statistical information of the locus.

## Verification using test data

## Test data

Our test data contains 36 samples initiated for external quality assessments with the University of California Los Angeles (UCLA) International HLA DNA Exchange (Los Angeles, CA, USA). Genomic DNAs with known HLA typing results were obtained from UCLA and amplified using locus-specific primers. All samples have been typed for HLA-A, -B, -C, -DRB1, and -DQB1 by Sanger sequencing using a 3730XL DNA Analyzer (Applied Biosystems, Foster City, CA). The sequencing reaction was performed using the BigDye® Terminator v3.1 Cycle Sequencing Ready Reaction Kit (Applied Biosystems). The sequencing reaction was performed using the BigDye® Terminator v3.1 Cycle Sequencing Ready Reaction Kit (Applied Biosystems). The Sanger sequencing strategy involved amplification spanning exons 2, 3, and 4 for HLA-A, -B, -C, and two separate amplicons for exons 2 and 3 for HLA-DQB1 and -DPB1 (Table S1). Group-specific sequencing primers (GSSP) were applied to resolve ambiguities. The test data have been deposited in the CNSA (https://db.cngb.org/cnsa/) of CNGBdb with an accession code CNP0000512.

## Results

The sequence was analyzed with SOAPTyping. The typing results were compared to the consensus based on the high resolution provided by UCLA. The consistency of SOAPTyping in typing HLA alleles at two-field designations was verified to be accurate at the level of 100% (36/36) for HLA-A, -B, -C, -DR and -DQ. The detailed results of 36 tested samples were shown in Table S8. The means of these columns are sample name from UCLA, Population of the sample, Allele1 type of the Gene HLA-A, Allele2 type of the Gene HLA-A, Allele1 type of the Gene HLA-B, Allele2 type of the Gene HLA-B, Allele1 type of the Gene HLA-C, Allele2 type of the Gene HLA-C, Allele1 type of the Gene HLA-DRB1, Allele2 type of the Gene HLA-DRB1, Allele1 type of the Gene HLA-DQB1, Allele2 type of the Gene HLA-DQB.

Table S8. SOAPTyping results of 36 samples from UCLA International DNA Exchange

| SampleNameUcla | Population | A1 | A2 | B1 | B2 | C1 | C2 | DRB1 | DRB2 | DQB1 | DQB2 |
| --- | --- | --- | --- | --- | --- | --- | --- | --- | --- | --- | --- |
| #867 | BLACK | A*02:01 | A*66:02 | B*44:02 | B*57:03 | C*05:01 | C*06:02 | DRB1*03:01 | DRB1*04:02 | DQB1*02:01 | DQB1*03:02 |
| #868 | HISPANIC | A*01:01 | A*02:01 | B*52:01 | B*57:01 | C*03:03 | C*07:01 | DRB1*01:01 | DRB1*14:06 | DQB1*05:01 | DQB1*03:01 |
| #869 | ASIAN | A*02:01 | A*31:01 | B*27:05 | B*40:06 | C*02:02 | C*08:01 | DRB1*04:01 | DRB1*09:01 | DQB1*03:02 | DQB1*03:03 |
| #870 | CAUCASIAN | A*11:01 | A*25:01 | B*15:01 | B*44:02 | C*03:03 | C*05:01 | DRB1*04:01 | DRB1*13:01 | DQB1*06:03 | DQB1*03:01 |
| #871 | HISPANIC | A*26:01 | A*29:02 | B*27:05 | B*44:03 | C*02:02 | C*16:01 | DRB1*07:01 | DRB1*08:03 | DQB1*02:02 | DQB1*03:01 |
| #872 | CAUCASIAN | A*03:01 | A*03:01 | B*07:02 | B*44:02 | C*05:01 | C*07:02 | DRB1*04:01 | DRB1*15:01 | DQB1*06:02 | DQB1*03:01 |
| #873 | HISPANIC | A*03:01 | A*03:01 | B*07:02 | B*45:01 | C*06:02 | C*07:02 | DRB1*04:01 | DRB1*16:01 | DQB1*05:02 | DQB1*03:01 |
| #874 | ASIAN | A*02:07 | A*11:01 | B*15:58 | B*46:01 | C*01:02 | C*01:02 | DRB1*14:54 | DRB1*15:01 | DQB1*05:02 | DQB1*05:02 |
| #875 | CAUCASIAN | A*02:01 | A*30:02 | B*15:04 | B*18:01 | C*03:03 | C*05:01 | DRB1*03:01 | DRB1*08:04 | DQB1*02:01 | DQB1*04:02 |
| #876 | CAUCASIAN | A*02:01 | A*66:01 | B*27:05 | B*40:01 | C*01:02 | C*03:04 | DRB1*01:01 | DRB1*04:04 | DQB1*05:01 | DQB1*03:02 |
| #877 | BLACK | A*24:02 | A*34:02 | B*13:02 | B*14:02 | C*06:02 | C*08:02 | DRB1*07:01 | DRB1*14:54 | DQB1*05:03 | DQB1*02:02 |
| #878 | UNKNOWN | A*26:01 | A*26:03 | B*40:02 | B*51:01 | C*03:04 | C*14:02 | DRB1*09:01 | DRB1*14:03 | DQB1*03:01 | DQB1*03:03 |
| #879 | CAUCASIAN | A*02:01 | A*26:01 | B*14:01 | B*55:01 | C*03:03 | C*08:02 | DRB1*07:01 | DRB1*11:03 | DQB1*02:02 | DQB1*03:01 |
| #880 | HISPANIC | A*02:01 | A*26:01 | B*14:02 | B*53:01 | C*01:02 | C*08:02 | DRB1*03:01 | DRB1*13:02 | DQB1*06:09 | DQB1*02:01 |
| #881 | BLACK | A*03:01 | A*33:01 | B*51:01 | B*57:03 | C*01:02 | C*18:02 | DRB1*11:01 | DRB1*11:01 | DQB1*03:01 | DQB1*03:19 |
| #882 | HISPANIC | A*02:01 | A*02:06 | B*07:02 | B*49:01 | C*07:01 | C*07:02 | DRB1*13:02 | DRB1*15:01 | DQB1*06:02 | DQB1*06:04 |
| #883 | ASIAN | A*02:06 | A*24:02 | B*56:01 | B*56:02 | C*01:02 | C*01:02 | DRB1*04:03 | DRB1*09:01 | DQB1*03:02 | DQB1*03:03 |
| #884 | CAUCASIAN | A*29:02 | A*32:01 | B*13:02 | B*44:03 | C*06:02 | C*16:01 | DRB1*07:01 | DRB1*12:01 | DQB1*02:02 | DQB1*03:01 |
| #885 | BLACK | A*25:01 | A*74:01 | B*08:01 | B*15:03 | C*02:10 | C*07:01 | DRB1*03:01 | DRB1*11:01 | DQB1*02:01 | DQB1*03:19 |
| #886 | HISPANIC | A*01:01 | A*25:01 | B*51:01 | B*57:01 | C*06:02 | C*12:03 | DRB1*04:07 | DRB1*13:02 | DQB1*06:04 | DQB1*03:01 |
| #887 | HISPANIC | A*02:01 | A*34:02 | B*40:01 | B*53:01 | C*03:04 | C*06:02 | DRB1*09:01 | DRB1*11:01 | DQB1*02:02 | DQB1*03:01 |
| #888 | ASIAN | A*02:01 | A*02:06 | B*40:01 | B*55:02 | C*04:82 | C*08:01 | DRB1*04:03 | DRB1*08:03 | DQB1*06:01 | DQB1*03:02 |
| #889 | CAUCASIAN | A*01:01 | A*29:02 | B*08:01 | B*45:01 | C*06:02 | C*07:01 | DRB1*04:01 | DRB1*04:01 | DQB1*03:01 | DQB1*03:02 |
| #890 | CAUCASIAN | A*03:01 | A*30:02 | B*14:02 | B*35:01 | C*04:01 | C*08:02 | DRB1*08:06 | DRB1*13:01 | DQB1*06:02 | DQB1*06:03 |
| #891 | BLACK | A*66:02 | A*68:02 | B*39:10 | B*57:03 | C*12:03 | C*18:02 | DRB1*13:02 | DRB1*16:02 | DQB1*05:02 | DQB1*06:09 |
| #892 | HISPANIC | A*02:06 | A*31:01 | B*15:01 | B*39:06 | C*01:02 | C*07:02 | DRB1*08:02 | DRB1*14:06 | DQB1*03:01 | DQB1*04:02 |
| #893 | HISPANIC | A*23:01 | A*30:10 | B*41:01 | B*41:01 | C*06:02 | C*08:02 | DRB1*04:05 | DRB1*11:01 | DQB1*06:02 | DQB1*02:02 |
| #894 | CAUCASIAN | A*01:01 | A*29:02 | B*07:02 | B*07:02 | C*07:02 | C*07:02 | DRB1*15:01 | DRB1*15:01 | DQB1*06:02 | DQB1*06:02 |
| #895 | ASIAN | A*02:01 | A*02:06 | B*40:06 | B*51:01 | C*08:01 | C*15:02 | DRB1*08:02 | DRB1*08:03 | DQB1*06:01 | DQB1*04:02 |
| #896 | CAUCASIAN | A*02:01 | A*03:01 | B*27:02 | B*41:01 | C*02:02 | C*17:01 | DRB1*04:04 | DRB1*13:01 | DQB1*06:03 | DQB1*04:02 |
| #897 | HISPANIC | A*02:05 | A*68:01 | B*48:01 | B*50:01 | C*06:02 | C*08:01 | DRB1*04:04 | DRB1*07:01 | DQB1*02:02 | DQB1*03:02 |
| #898 | BLACK | A*02:01 | A*24:02 | B*14:01 | B*45:01 | C*02:10 | C*16:01 | DRB1*01:02 | DRB1*07:01 | DQB1*05:01 | DQB1*02:02 |
| #899 | UNKNOWN | A*03:01 | A*33:03 | B*18:01 | B*49:01 | C*05:01 | C*07:01 | DRB1*03:02 | DRB1*11:02 | DQB1*03:19 | DQB1*04:02 |
| #900 | CAUCASIAN | A*01:01 | A*02:01 | B*27:05 | B*37:01 | C*02:02 | C*06:02 | DRB1*10:01 | DRB1*14:54 | DQB1*05:01 | DQB1*05:03 |
| #901 | ASIAN | A*03:01 | A*03:01 | B*07:02 | B*35:01 | C*04:01 | C*07:02 | DRB1*01:01 | DRB1*15:01 | DQB1*05:01 | DQB1*06:02 |
| #902 | CAUCASIAN | A*01:01 | A*02:01 | B*08:01 | B*37:01 | C*06:02 | C*07:01 | DRB1*03:01 | DRB1*11:01 | DQB1*02:01 | DQB1*03:01 |

We also test 100 clinical samples using both SOAPTyping and uTYPE, and the results are consistent between these two tools. The detailed results of 100 tested samples were shown in Table S9. The means of these columns are sample name, Allele1 type of the Gene HLA-A, Allele2 type of the Gene HLA-A, Allele1 type of the Gene HLA-B, Allele2 type of the Gene HLA-B, Allele1 type of the Gene HLA-C, Allele2 type of the Gene HLA-C, Allele1 type of the Gene HLA-DRB1, Allele2 type of the Gene HLA-DRB1, Allele1 type of the Gene HLA-DQB1, Allele2 type of the Gene HLA-DQB.

Table S9. The HLA typing results of 100 clinical samples

| SampleName | A | A | B | B | DRB1 | DRB1 | C | C | DQB1 | DQB1 |
| --- | --- | --- | --- | --- | --- | --- | --- | --- | --- | --- |
| Sample1 | A*02:07 | A*24:33 | B*46:01 | B*51:06 | DRB1*09:01 | DRB1*12:02 | C*01:02 | C*12:04 | DQB1*05:02 | DQB1*03:03 |
| Sample2 | A*01:01 | A*30:01 | B*13:02 | B*52:01 | DRB1*11:03 | DRB1*15:02 | C*06:02 | C*12:02 | DQB1*06:01 | DQB1*03:01 |
| Sample3 | A*02:01 | A*02:06 | B*35:01 | B*35:12 | DRB1*08:02 | DRB1*16:02 | C*04:01 | C*04:01 | DQB1*03:01 | DQB1*04:02 |
| Sample4 | A*02:01 | A*30:01 | B*42:01 | B*49:01 | DRB1*11:01 | DRB1*13:03 | C*07:01 | C*17:01 | DQB1*06:02 | DQB1*02:02 |
| Sample5 | A*02:06 | A*30:02 | B*27:05 | B*40:02 | DRB1*08:02 | DRB1*15:01 | C*02:02 | C*03:06 | DQB1*06:02 | DQB1*04:02 |
| Sample6 | A*02:01 | A*26:01 | B*27:05 | B*40:01 | DRB1*08:01 | DRB1*11:01 | C*01:02 | C*03:04 | DQB1*03:01 | DQB1*04:02 |
| Sample7 | A*11:01 | A*25:01 | B*18:01 | B*51:01 | DRB1*07:01 | DRB1*09:01 | C*12:03 | C*15:02 | DQB1*02:02 | DQB1*03:03 |
| Sample8 | A*24:02 | A*34:01 | B*13:01 | B*40:10 | DRB1*04:03 | DRB1*04:05 | C*04:03 | C*12:03 | DQB1*03:02 | DQB1*04:02 |
| Sample9 | A*02:01 | A*23:01 | B*35:12 | B*41:01 | DRB1*07:01 | DRB1*08:02 | C*04:01 | C*17:01 | DQB1*02:02 | DQB1*04:02 |
| Sample10 | A*02:01 | A*24:02 | B*07:02 | B*18:01 | DRB1*03:01 | DRB1*15:01 | C*05:01 | C*15:02 | DQB1*06:02 | DQB1*02:01 |
| Sample11 | A*01:01 | A*03:01 | B*07:02 | B*57:01 | DRB1*07:01 | DRB1*11:01 | C*06:02 | C*07:02 | DQB1*03:01 | DQB1*03:03 |
| Sample12 | A*02:01 | A*02:01 | B*15:01 | B*52:01 | DRB1*04:01 | DRB1*13:01 | C*03:04 | C*16:01 | DQB1*06:03 | DQB1*03:02 |
| Sample13 | A*02:01 | A*24:02 | B*15:15 | B*15:39 | DRB1*04:04 | DRB1*04:04 | C*01:02 | C*03:03 | DQB1*03:02 | DQB1*03:02 |
| Sample14 | A*02:01 | A*29:02 | B*40:01 | B*44:03 | DRB1*07:01 | DRB1*13:02 | C*03:04 | C*16:01 | DQB1*06:04 | DQB1*02:02 |
| Sample15 | A*01:01 | A*03:01 | B*08:01 | B*52:01 | DRB1*01:01 | DRB1*03:01 | C*04:01 | C*07:01 | DQB1*05:01 | DQB1*02:01 |
| Sample16 | A*23:01 | A*30:01 | B*08:01 | B*53:01 | DRB1*13:04 | DRB1*15:03 | C*03:04 | C*04:01 | DQB1*06:02 | DQB1*03:19 |
| Sample17 | A*11:01 | A*68:03 | B*27:05 | B*39:05 | DRB1*01:01 | DRB1*04:07 | C*01:02 | C*07:02 | DQB1*05:01 | DQB1*03:02 |
| Sample18 | A*11:01 | A*24:02 | B*15:35 | B*35:01 | DRB1*15:01 | DRB1*15:02 | C*07:02 | C*12:03 | DQB1*05:02 | DQB1*05:02 |
| Sample19 | A*24:02 | A*26:01 | B*15:01 | B*15:09 | DRB1*04:03 | DRB1*04:04 | C*01:02 | C*07:04 | DQB1*03:01 | DQB1*03:02 |
| Sample20 | A*02:02 | A*29:02 | B*44:03 | B*81:01 | DRB1*07:01 | DRB1*12:01 | C*08:04 | C*16:01 | DQB1*05:01 | DQB1*02:02 |
| Sample21 | A*02:13 | A*03:01 | B*07:02 | B*40:02 | DRB1*07:01 | DRB1*14:02 | C*03:04 | C*07:02 | DQB1*03:01 | DQB1*03:03 |
| Sample22 | A*02:01 | A*33:03 | B*35:01 | B*52:01 | DRB1*10:01 | DRB1*13:03 | C*04:01 | C*16:01 | DQB1*05:01 | DQB1*03:01 |
| Sample23 | A*01:01 | A*24:02 | B*44:03 | B*51:01 | DRB1*11:01 | DRB1*15:01 | C*07:06 | C*15:04 | DQB1*06:02 | DQB1*03:01 |
| Sample24 | A*01:01 | A*33:03 | B*27:05 | B*40:01 | DRB1*04:05 | DRB1*15:01 | C*02:02 | C*07:02 | DQB1*06:02 | DQB1*03:02 |
| Sample25 | A*02:01 | A*66:02 | B*44:02 | B*57:03 | DRB1*03:01 | DRB1*04:02 | C*05:01 | C*06:02 | DQB1*02:01 | DQB1*03:02 |
| Sample26 | A*01:01 | A*02:01 | B*52:01 | B*57:01 | DRB1*01:01 | DRB1*14:06 | C*03:03 | C*07:01 | DQB1*05:01 | DQB1*03:01 |
| Sample27 | A*02:01 | A*31:01 | B*27:05 | B*40:06 | DRB1*04:01 | DRB1*09:01 | C*02:02 | C*08:01 | DQB1*03:02 | DQB1*03:03 |
| Sample28 | A*11:01 | A*25:01 | B*15:01 | B*44:02 | DRB1*04:01 | DRB1*13:01 | C*03:03 | C*05:01 | DQB1*06:03 | DQB1*03:01 |
| Sample29 | A*26:01 | A*29:02 | B*27:05 | B*44:03 | DRB1*07:01 | DRB1*08:03 | C*02:02 | C*16:01 | DQB1*02:02 | DQB1*03:01 |
| Sample30 | A*03:01 | A*03:01 | B*07:02 | B*44:02 | DRB1*04:01 | DRB1*15:01 | C*05:01 | C*07:02 | DQB1*06:02 | DQB1*03:01 |
| Sample31 | A*01:01 | A*30:01 | B*13:02 | B*41:01 | DRB1*07:01 | DRB1*13:14 | C*06:02 | C*17:01 | DQB1*02:02 | DQB1*03:01 |
| Sample32 | A*02:05 | A*03:05 | B*15:02 | B*50:01 | DRB1*03:01 | DRB1*04:03 | C*06:02 | C*08:01 | DQB1*02:01 | DQB1*03:02 |
| Sample33 | A*02:01 | A*33:03 | B*15:18 | B*58:01 | DRB1*04:38 | DRB1*07:01 | C*03:02 | C*07:04 | DQB1*02:02 | DQB1*03:01 |
| Sample34 | A*24:33 | A*30:01 | B*13:02 | B*40:02 | DRB1*07:01 | DRB1*08:03 | C*03:03 | C*06:02 | DQB1*06:01 | DQB1*02:02 |
| Sample35 | A*02:01 | A*02:24 | B*40:02 | B*52:01 | DRB1*13:02 | DRB1*15:01 | C*03:04 | C*12:02 | DQB1*06:02 | DQB1*06:04 |
| Sample36 | A*02:01 | A*02:01 | B*40:01 | B*44:05 | DRB1*15:01 | DRB1*16:01 | C*02:02 | C*03:04 | DQB1*05:02 | DQB1*06:02 |
| Sample37 | A*11:01 | A*26:01 | B*13:01 | B*27:04 | DRB1*08:03 | DRB1*14:54 | C*03:04 | C*15:09 | DQB1*05:03 | DQB1*06:01 |
| Sample38 | A*02:XX | A*24:02 | B*46:01 | B*48:01 | DRB1*01:01 | DRB1*04:17 | C*01:02 | C*08:22 | DQB1*05:01 | DQB1*04:01 |
| Sample39 | A*02:01 | A*11:01 | B*40:01 | B*40:06 | DRB1*11:01 | DRB1*11:12 | C*07:02 | C*08:22 | DQB1*03:01 | DQB1*03:01 |
| Sample40 | A*03:01 | A*24:02 | B*15:16 | B*40:02 | DRB1*13:02 | DRB1*16:02 | C*04:01 | C*14:02 | DQB1*05:02 | DQB1*06:04 |
| Sample41 | A*33:03 | A*68:02 | B*39:10 | B*44:03 | DRB1*13:02 | DRB1*13:02 | C*12:03 | C*14:03 | DQB1*06:04 | DQB1*06:09 |
| Sample42 | A*02:74 | A*11:01 | B*07:02 | B*51:01 | DRB1*01:01 | DRB1*12:10 | C*07:02 | C*14:02 | DQB1*05:01 | DQB1*03:01 |
| Sample43 | A*02:06 | A*31:01 | B*15:01 | B*35:01 | DRB1*14:05 | DRB1*15:01 | C*03:03 | C*03:03 | DQB1*05:03 | DQB1*06:02 |
| Sample44 | A*02:01 | A*02:07 | B*46:01 | B*51:05 | DRB1*01:01 | DRB1*12:01 | C*01:02 | C*04:01 | DQB1*05:01 | DQB1*03:01 |
| Sample45 | A*02:07 | A*02:20 | B*13:01 | B*44:02 | DRB1*04:01 | DRB1*11:04 | C*03:04 | C*05:01 | DQB1*03:01 | DQB1*03:01 |
| Sample46 | A*02:01 | A*11:01 | B*51:01 | B*55:12 | DRB1*07:01 | DRB1*13:02 | C*01:02 | C*14:02 | DQB1*06:09 | DQB1*02:02 |
| Sample47 | A*02:12 | A*31:01 | B*13:01 | B*40:06 | DRB1*09:01 | DRB1*12:02 | C*03:04 | C*08:01 | DQB1*03:01 | DQB1*03:03 |
| Sample48 | A*03:01 | A*24:02 | B*15:18 | B*44:02 | DRB1*13:01 | DRB1*14:03 | C*05:01 | C*08:01 | DQB1*06:03 | DQB1*03:01 |
| Sample49 | A*02:01 | A*11:01 | B*15:02 | B*15:129 | DRB1*15:01 | DRB1*15:01 | C*07:02 | C*08:01 | DQB1*06:01 | DQB1*06:02 |
| Sample50 | A*02:07 | A*36:02 | B*46:01 | B*57:01 | DRB1*09:01 | DRB1*13:02 | C*01:02 | C*06:02 | DQB1*06:04 | DQB1*03:03 |
| Sample51 | A*01:01 | A*24:02 | B*15:09 | B*57:01 | DRB1*01:01 | DRB1*07:01 | C*06:02 | C*07:04 | DQB1*05:01 | DQB1*03:03 |
| Sample52 | A*02:01 | A*24:02 | B*35:01 | B*40:48 | DRB1*08:03 | DRB1*15:01 | C*03:03 | C*07:02 | DQB1*06:01 | DQB1*06:02 |
| Sample53 | A*24:02 | A*29:01 | B*07:05 | B*40:06 | DRB1*10:01 | DRB1*12:02 | C*08:22 | C*15:29 | DQB1*05:01 | DQB1*03:01 |
| Sample54 | A*02:05 | A*30:01 | B*13:02 | B*15:18 | DRB1*07:01 | DRB1*11:19 | C*06:02 | C*07:04 | DQB1*02:02 | DQB1*03:01 |
| Sample55 | A*02:01 | A*33:03 | B*35:11 | B*58:01 | DRB1*13:02 | DRB1*15:01 | C*03:02 | C*03:03 | DQB1*06:02 | DQB1*06:09 |
| Sample56 | A*31:01 | A*74:03 | B*35:01 | B*58:01 | DRB1*03:01 | DRB1*12:01 | C*03:02 | C*04:01 | DQB1*02:01 | DQB1*03:01 |
| Sample57 | A*24:07 | A*74:02 | B*15:12 | B*35:01 | DRB1*09:01 | DRB1*12:02 | C*03:03 | C*03:03 | DQB1*05:02 | DQB1*03:03 |
| Sample58 | A*02:01 | A*30:01 | B*13:02 | B*15:21 | DRB1*07:01 | DRB1*15:02 | C*04:03 | C*06:02 | DQB1*06:01 | DQB1*02:02 |
| Sample59 | A*26:01 | A*30:01 | B*48:01 | B*52:01 | DRB1*14:05 | DRB1*14:10 | C*07:02 | C*08:03 | DQB1*05:03 | DQB1*05:10 |
| Sample60 | A*02:131 | A*24:02 | B*40:06 | B*40:06 | DRB1*10:01 | DRB1*14:04 | C*03:04 | C*14:02 | DQB1*05:01 | DQB1*05:03 |
| Sample61 | A*01:01 | A*33:03 | B*52:01 | B*57:01 | DRB1*07:01 | DRB1*08:10 | C*06:02 | C*07:02 | DQB1*02:02 | DQB1*03:01 |
| Sample62 | A*23:01 | A*30:10 | B*13:02 | B*18:01 | DRB1*13:02 | DRB1*13:02 | C*05:01 | C*06:02 | DQB1*06:04 | DQB1*06:04 |
| Sample63 | A*02:01 | A*03:01 | B*35:03 | B*44:27 | DRB1*14:54 | DRB1*16:01 | C*04:01 | C*07:04 | DQB1*05:02 | DQB1*05:03 |
| Sample64 | A*03:01 | A*24:02 | B*08:01 | B*35:01 | DRB1*03:01 | DRB1*04:05 | C*03:03 | C*03:04 | DQB1*02:01 | DQB1*04:01 |
| Sample65 | A*02:01 | A*02:03 | B*15:39 | B*40:01 | DRB1*14:05 | DRB1*15:01 | C*03:03 | C*03:04 | DQB1*05:03 | DQB1*06:02 |
| Sample66 | A*11:04 | A*24:02 | B*15:01 | B*40:01 | DRB1*04:06 | DRB1*11:01 | C*03:03 | C*03:04 | DQB1*03:01 | DQB1*03:02 |
| Sample67 | A*11:01 | A*24:02 | B*15:27 | B*54:01 | DRB1*11:01 | DRB1*14:05 | C*01:02 | C*04:01 | DQB1*05:03 | DQB1*03:01 |
| Sample68 | A*02:08 | A*26:01 | B*27:05 | B*50:01 | DRB1*01:01 | DRB1*04:02 | C*01:02 | C*06:02 | DQB1*05:01 | DQB1*03:02 |
| Sample69 | A*26:08 | A*30:01 | B*13:02 | B*51:01 | DRB1*04:01 | DRB1*07:01 | C*06:02 | C*15:02 | DQB1*02:02 | DQB1*03:02 |
| Sample70 | A*11:01 | A*24:02 | B*27:03 | B*40:01 | DRB1*03:01 | DRB1*04:03 | C*03:03 | C*07:02 | DQB1*02:01 | DQB1*03:02 |
| Sample71 | A*31:01 | A*33:03 | B*18:01 | B*40:02 | DRB1*08:03 | DRB1*11:01 | C*03:04 | C*12:05 | DQB1*06:01 | DQB1*03:01 |
| Sample72 | A*01:01 | A*02:07 | B*27:24 | B*57:01 | DRB1*04:05 | DRB1*13:01 | C*06:02 | C*14:02 | DQB1*06:03 | DQB1*04:01 |
| Sample73 | A*11:02 | A*24:02 | B*27:15 | B*40:01 | DRB1*14:05 | DRB1*14:05 | C*03:03 | C*12:02 | DQB1*05:03 | DQB1*05:03 |
| Sample74 | A*30:01 | A*33:03 | B*13:02 | B*58:01 | DRB1*11:01 | DRB1*13:02 | C*03:36 | C*06:02 | DQB1*06:09 | DQB1*03:01 |
| Sample75 | A*11:01 | A*31:01 | B*13:02 | B*15:38 | DRB1*07:01 | DRB1*11:01 | C*03:03 | C*06:02 | DQB1*02:02 | DQB1*03:01 |
| Sample76 | A*02:17 | A*24:02 | B*15:01 | B*40:01 | DRB1*11:01 | DRB1*12:02 | C*03:03 | C*15:02 | DQB1*03:01 | DQB1*03:01 |
| Sample77 | A*26:01 | A*33:03 | B*40:02 | B*44:03 | DRB1*12:02 | DRB1*15:01 | C*03:03 | C*14:03 | DQB1*06:11 | DQB1*03:01 |
| Sample78 | A*11:01 | A*24:02 | B*35:04 | B*54:01 | DRB1*11:01 | DRB1*14:05 | C*01:02 | C*03:03 | DQB1*05:03 | DQB1*03:01 |
| Sample79 | A*02:06 | A*03:01 | B*40:06 | B*51:01 | DRB1*11:01 | DRB1*11:04 | C*08:01 | C*15:04 | DQB1*03:01 | DQB1*03:01 |
| Sample80 | A*02:01 | A*11:02 | B*07:02 | B*40:01 | DRB1*12:02 | DRB1*14:05 | C*07:02 | C*07:154 | DQB1*05:03 | DQB1*03:01 |
| Sample81 | A*31:01 | A*31:01 | B*15:58 | B*51:01 | DRB1*09:01 | DRB1*14:54 | C*01:02 | C*14:02 | DQB1*05:02 | DQB1*03:03 |
| Sample82 | A*02:01 | A*11:01 | B*35:01 | B*40:01 | DRB1*12:01 | DRB1*15:06 | C*04:01 | C*07:02 | DQB1*05:02 | DQB1*03:17 |
| Sample83 | A*02:06 | A*11:01 | B*15:46 | B*58:01 | DRB1*07:01 | DRB1*15:01 | C*03:02 | C*03:03 | DQB1*06:02 | DQB1*03:03 |
| Sample84 | A*03:01 | A*11:01 | B*38:01 | B*40:01 | DRB1*12:02 | DRB1*14:54 | C*07:02 | C*12:03 | DQB1*05:03 | DQB1*03:01 |
| Sample85 | A*26:20 | A*30:01 | B*13:02 | B*35:01 | DRB1*01:01 | DRB1*07:01 | C*04:69 | C*06:02 | DQB1*05:01 | DQB1*02:02 |
| Sample86 | A*11:01 | A*68:01 | B*40:01 | B*44:03 | DRB1*07:13 | DRB1*14:05 | C*03:03 | C*07:06 | DQB1*05:03 | DQB1*02:02 |
| Sample87 | A*02:01 | A*68:02 | B*14:02 | B*15:01 | DRB1*01:02 | DRB1*09:01 | C*08:02 | C*15:02 | DQB1*05:01 | DQB1*03:03 |
| Sample88 | A*02:01 | A*26:01 | B*27:36 | B*35:01 | DRB1*14:54 | DRB1*15:01 | C*03:03 | C*03:04 | DQB1*05:02 | DQB1*06:01 |
| Sample89 | A*11:01 | A*68:24 | B*35:08 | B*50:01 | DRB1*07:01 | DRB1*12:02 | C*05:01 | C*06:02 | DQB1*02:02 | DQB1*03:01 |
| Sample90 | A*02:01 | A*31:13 | B*40:01 | B*40:03 | DRB1*11:01 | DRB1*15:01 | C*03:04 | C*03:04 | DQB1*06:02 | DQB1*03:01 |
| Sample91 | A*11:01 | A*26:01 | B*07:02 | B*27:05 | DRB1*01:01 | DRB1*04:04 | C*02:02 | C*07:67 | DQB1*05:01 | DQB1*03:02 |
| Sample92 | A*01:01 | A*33:03 | B*51:06 | B*58:01 | DRB1*03:01 | DRB1*15:02 | C*03:02 | C*12:04 | DQB1*06:01 | DQB1*02:01 |
| Sample93 | A*03:01 | A*26:03 | B*15:01 | B*27:05 | DRB1*04:01 | DRB1*15:01 | C*02:02 | C*03:03 | DQB1*06:02 | DQB1*03:01 |
| Sample94 | A*02:10 | A*31:06 | B*40:06 | B*44:02 | DRB1*01:01 | DRB1*11:01 | C*08:01 | C*15:02 | DQB1*05:01 | DQB1*03:01 |
| Sample95 | A*02:01 | A*03:01 | B*15:01 | B*44:03 | DRB1*01:01 | DRB1*03:01 | C*01:02 | C*04:01 | DQB1*05:01 | DQB1*02:01 |
| Sample96 | A*26:01 | A*68:03 | B*14:01 | B*35:48 | DRB1*04:07 | DRB1*07:01 | C*07:02 | C*08:02 | DQB1*02:02 | DQB1*03:02 |
| Sample97 | A*33:03 | A*74:01 | B*49:01 | B*51:01 | DRB1*13:03 | DRB1*15:03 | C*07:01 | C*16:01 | DQB1*06:02 | DQB1*03:01 |
| Sample98 | A*02:01 | A*02:05 | B*35:17 | B*58:01 | DRB1*04:08 | DRB1*13:02 | C*04:01 | C*07:18 | DQB1*06:09 | DQB1*03:02 |
| Sample99 | A*31:01 | A*68:01 | B*35:12 | B*45:01 | DRB1*04:05 | DRB1*08:02 | C*04:01 | C*06:02 | DQB1*03:02 | DQB1*04:02 |
| Sample100 | A*01:01 | A*01:01 | B*55:01 | B*57:01 | DRB1*08:03 | DRB1*11:04 | C*03:03 | C*06:02 | DQB1*03:01 | DQB1*03:01 |

The analysis time had also been recorded in 10 samples/run.

Table S10. The running time of 100 clinical samples.

| run | 1 | 2 | 3 | 4 | 5 | 6 | 7 | 8 | 9 | 10 | Sum |
| --- | --- | --- | --- | --- | --- | --- | --- | --- | --- | --- | --- |
| SOAPTyping running time (seconds) | | | | | | | | | | | |
| A | 23s | 34s | 24s | 20s | 16s | 19s | 16s | 17s | 16s | 15s | 200 |
| B | 28s | 20s | 24s | 22s | 17s | 16s | 20s | 16s | 17s | 16s | 196 |
| C | 30s | 32s | 26s | 28s | 16s | 17s | 14s | 15s | 16s | 16s | 210 |
| DRB1 | 14s | 13s | 13s | 15s | 10s | 7s | 7s | 6s | 10s | 6s | 101 |
| DQB1 | 18s | 23s | 16s | 16s | 10s | 11s | 10s | 11s | 11s | 10s | 136 |
| Sum | **113** | **122** | **103** | **101** | **69** | **70** | **67** | **65** | **70** | **63** | **843** |
| uTYPE running time (seconds) | | | | | | | | | | | |
| A | 125s | 136s | 156s | 124s | 104s | 62s | 83s | 92s | 73s | 82s | 1037 |
| B | 168s | 253s | 242s | 219s | 213s | 169s | 177s | 185s | 183s | 244s | 2053 |
| C | 113s | 153s | 138s | 131s | 120s | 124s | 122s | 125s | 138s | 120s | 1284 |
| DRB1 | 20s | 18s | 16s | 21s | 18s | 18s | 20s | 15s | 13s | 21s | 180 |
| DQB1 | 10s | 10s | 10s | 9s | 7s | 8s | 5s | 8s | 8s | 9s | 84 |
| Sum | **436** | **570** | **562** | **504** | **462** | **381** | **407** | **425** | **415** | **476** | **4638** |
